# From Genetics to Epigenetics: Top 4 Aspects for Improved SARS-CoV-2 Vaccine Designs as Paradigmatic Examples

**DOI:** 10.1055/s-0041-1739495

**Published:** 2021-11-09

**Authors:** Darja Kanduc

**Affiliations:** 1 Department of Biosciences, Biotechnologies and Biopharmaceutics, University of Bari, Bari, Italy

**Keywords:** vaccines, immunization, (epi) genetic factors

## Abstract

This literature review described the genetic and biochemical factors that may have been overlooked in the formulation of vaccines and that most likely underlie possible issues with mass vaccination.

## Introduction

Vaccines are a main tool of current global health strategies against infectious agents. Continuously improving vaccine formulation and designs is therefore of crucial importance in light of current global events. As a cautionary tale, one can take the recent dengue vaccine, 1 and, to a certain extent, the suboptimal results of the global immunization campaign against SARS-CoV-2. After the seemingly huge initial success of the worldwide SARS-CoV-2 vaccination, the virus is still spreading across the human population, and the active immunization might not confer adequate protection against new variants. 2 Why have such intense scientific and economic efforts produced suboptimal results? Searching for answers, this review explored the possible factors to keep producing better and safer vaccines on a mass scale.

## Fact 1: Molecular Mimicry between Microbial and Human Proteins can Lead to Cross-reactivity

Microbial proteins are mostly composed of peptide sequences that are also present in human proteins. 3 4 A consequence of such a peptide sharing is that cross-reactive autoantibodies (AAbs) can be generated following exposure to infectious agents by infection or vaccination. 5 Indeed, if antibodies against a pathogen protein hit sequences that are also present in human proteins, then it is logical to conclude that hitting the pathogen protein might also imply the possibility of targeting human proteins. Depending on the number and functions of the targeted human proteins, various clinical consequences might occur. 5 Therefore, an intrinsic property of vaccines based on full-length pathogen proteins is their capability of inducing harmful AAbs that cross-react with human proteins, thus possibly causing diseases in the human host. Said with a metaphor, hitting the infectious enemy might cause collateral damages as well.

SARS-CoV-2 vaccines are no exception. As a matter of fact, SARS-CoV-2 proteins consist of peptide fragments that repeatedly occur and recur throughout human proteins, with only a part of them being exclusively present in the viral antigens and absent in the human proteome. 6 Hence, it is a noteworthy fact that adaptive humoral immune response to infection/vaccination directly correlates with severe diseases, also known as Coronavirus Disease 2019 (COVID-19), in symptomatic SARS-CoV-2 infection. 7 8 9 10 11 Indeed, COVID-19 appears to be largely an autoimmune disease 12 with molecular mimicry as a crucial mechanism suspected to drive autoimmunity. 6 13

COVID-19 comprehends a wide spectrum of disorders 6 13 including the following:

- Thromboses and hemostasis diseases.
- Pneumonia and pulmonary hypertension.
- Lymphomas and cancer of the lung and other organs.
- Cardiovascular disorders and sudden death.
- Multisystem inflammatory syndromes.
- Skin leukocytoclasia, hyperkeratosis, and parakeratosis.
- Neurodegeneration and neurological disorders from memory impairment, disturbances of higher cognitive functions such as working memory and executive function, to temporal lobe epilepsy, schizophrenia, Alzheimer's disease, and Parkinson's disease, *inter alia* .

Such vast and heavy pathological cross-reactivity sequelae had already been foreseen in 2020 6 at the very beginning of the SARS-CoV-2 pandemic and have been lately confirmed. 14

## Fact 2: Codon Usage Controls Pathogen Latency and (re)Activation

The human body is home to thousands of microbial organisms that silently inhabit our organs. Such a regimen of often completely asymptomatic coexistence is ruled by the human codon usage that represents a basic frontline instrument of the innate immunity against infectious agents. 15 16 Human codon usage does not allow the translation of pathogen genes that are characterized by codon usages that do not conform to the human codon usage. 15 16 Hence, the following events occur in the human host: 1) the synthesis of pathogen proteins is inhibited, 2) the pathogen load is low in that the pathogen replication does not occur, and 3) the infection acquires a chronic latent asymptomatic status characterized by low or zero protein synthesis, without pathologic consequences. In fact, *in absentia* of pathogen proteins, immune responses and the consequent autoimmune cross-reactions cannot obviously occur.

Conversely, pathogen gene sequences that have been optimized for human preferred codons are efficiently translated to proteins in the human host, where they induce immune responses that, because of molecular mimicry, are mostly associated with cross-reactivity against human proteins. In brief, vaccine formulations based on codon optimization of pathogen genes increase pathogen replicative fitness and pathogen protein load in the human organism, thus inducing harmful autoimmune cross-reactive responses.

With regard to the anti-SARS-CoV-2 vaccines, codon-optimized sequences encoding full-length SARS-CoV-2 spike glycoprotein (gp) have been used, 17 18 so that the synthesis of the spike gp protein increased. Such a codon optimization with consequent increased protein synthesis can activate effective antispike gp immune responses after vaccination but potentially can also induce harmful autoimmune cross-reactions, leading to COVID-19. 6 13 Said with an additional metaphor, codon optimization is equivalent to opening the doors to COVID-19.

## Fact 3: Nonhuman Primates are Inadequate in Preclinical Tests

As already observed by Hogan, 19 the Rhesus macaque model is of limited utility in preclinical tests, while only mice might represent a correct animal model for testing immunotherapies to be used in humans. 20 21 In particular, preclinical animal trials based on nonhuman primates are inadequate to reveal potential autoimmune cross-reactions following infection or immunization, in that molecular mimicry is high between pathogens and *Homo sapiens* but not between pathogens and nonhuman primates. 22 23 As a consequence, autoimmune cross-reactions cannot occur in primates at the high extent they do in humans.

Coherently with such data, nonhuman primates infected with SARS-CoV-2 develop a mild infection resembling asymptomatic human infection. 24 Nevertheless, it has to be highlighted that nonhuman primates have been used in testing anti-SARS-CoV-2 vaccines, 18 24 25 26 whereas valid animal models had to be rats or mice, that is, animals with a level of molecular mimicry with pathogens comparable to the level of molecular mimicry present in humans. 22 23 To use a metaphor once more, using nonhuman primates in preclinical tests is a swimming test in an empty swimming pool.

## Fact 4: Are Monoclonal Antibodies Really Monoclonal?

**Fig. 1:**
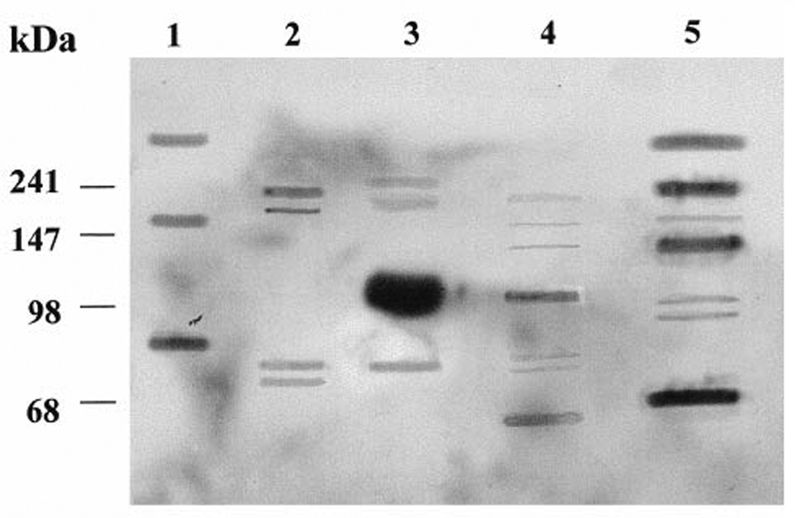
Anti-GD3 MAb MG22 cross-reacts with numerous proteins from cells of various origins. Lanes: 1) reticulocyte lysate; 2) wheat germ extract; 3) whole rat serum; 4) lymphoma cell lysate; 5) melanoma cell lysate. Molecular weight markers are on the left. (From Willers et al 32 , and further details therein).

By canonical definition, a monoclonal antibody (MAb) is an antibody that recognizes a unique antigenic epitope. In conflict, scientific research has documented that such MAb definition does not correspond to the reality, in that, *de facto* , MAbs are not exempt from cross-reactivity. As clearly proved already in 1981 by Dulbecco et al 27 and others, 28 29 30 31 32 33 34 35 36 37 38 cross-reactivity and multiple organ reactivity associate with MAbs. Here, a visual representation of the cross-reactivity burden that can associate with a MAb is offered in Fig. 1 . 32 Fig. 1 illustrates that MAb GD3, a MAb raised against the disialoganglioside GD3 melanoma antigen, cross-reacts with numerous melanoma proteins, the molecular weight of which range from 240 to 70 kDa ( Fig. 1 , lane 5). In addition, MAb MG22 also cross-reacts with proteins from a human lymphoblastoid cell line ( Fig. 1 , lane 4), which does not express GD3, and with proteins from normal cells of various origins ( Fig. 1 , lanes 1 to 3).

Such experimental scientific data 27 28 29 30 31 32 33 34 35 36 37 38 legitimate a crucial question: are MAbs really monoclonal and their effect predictable enough to be used in immunotherapies? Actually, MAbs might present unexpected consequences. Recent proposals for using MAbs to fight the current SARS-CoV-2 pandemic 39 40 must be weighed with extreme caution.

## Conclusion

Using SARS-CoV-2 infection/vaccination as a paradigmatic example and moving on from the etiology of the numerous diseases that can associate with infection, this review offers a unified theoretical basis for designing safe and more effective vaccines.

Indeed, from a scientific point of view, peptide sharing, that is, molecular mimicry, and the consequent potential cross-reactivity, provide the molecular platform and the basic mechanism that link infections to harmful autoimmunity, thereby supporting the concept of peptide uniqueness in designing safe immunotherapies exempt from cross-reactivity risks. As a matter of fact, since 1999, 32 it was suggested that only peptide motifs unique to the antigen of interest and absent in the human proteome have the potential to evoke safe, specific, and efficacious immune responses to fight infectious agents, cancer, and autoimmunity, 41 42 43 thus allowing for improved vaccine design and avoiding vaccinal failures. 1 44 45 46 47 48 49 50 51 52 53 54 55 56 57 58 59 60 61 62
